# Real-time cardiac phase contrast MRI blood flow including Valsalva and Mueller maneuver. Initial experiences

**DOI:** 10.1186/1532-429X-15-S1-E17

**Published:** 2013-01-30

**Authors:** Jan M Sohns, Christina Unterberg-Buchwald, Johannes T Kowallick, Michael Steinmetz, Christina Schulte, Wieland Staab, Arun Joseph, Klaus-Dietmar Merboldt, Dirk Voit, Shuo Zhang, Martin Uecker, Jens Frahm, Joachim Lotz

**Affiliations:** 1 Radiology, University Medical Center Georg-August-University, Göttingen, Germany; 2 Cardiology and Pneumology, University Medical Center Georg-August-University, Göttingen, Germany; 3 Pediatric Cardiology, University Medical Center Georg-August-University, Göttingen, Germany; 4 NMR Forschungs GmbH, Max-Planck-Institut für biophysikalische Chemie, Göttingen, Germany

## Background

A high resolution real-time phase-contrast MRI flow technique was used to measure flow dynamics in the ascending aorta as well as superior vena cava. MRI technique based on undersampled radial fast low-angle shot acquisitions with phase-sensitive image reconstructions by regularized nonlinear inversion. Normal flow values as well as flow measurements during physiologic stress tests like Valsalva (increased intrathoracic pressure) and Mueller (decreased intrathoracic pressure, reverse of Valsalva maneuver) were obtained in healthy volunteers.

## Methods

Blood flow was measured in the ascending aorta and superior vena cava using a single scan plane perpendicular to the ascending aorta at the level of the right pulmonary artery. In-plane resolution of 1.8 mm, section thickness of 6 mm at a real-time resolution of 48 ms was achieved by TR 3.44 ms; TE 2.76 ms; flip angle, 10 degrees and seven radial spokes per image. Scans were done in a clinical 3T scanner. ECG was co-registered for documentation only. Realtime scans were done for 20 seconds in normal measurements, 30 seconds for physiologic and stress maneuvers - 10 seconds normal breathing, 10 seconds applied stress, 10 seconds recovery time. Image analysis was done using a specifically modified prototype software Qflow by Medis, NL.

## Results

Realtime measurements were successful in all cases. We observed a decrease of blood-flow during Valsalva and Mueller maneuver in the ascending aorta. At the beginning of the maneuver, the blood flow increased under both increased and reduced intrathoracic pressure, followed by a continuous period of nearly 20 sec of decreased flow. A reactive hyperdynamic flow response was detected after restarting regular breathing.

## Conclusions

High resolution real-time MRI flow measurements are able to resolve detailed physiological blood flow changes during Vasalva and Mueller maneuvers with high reliability. The results are in consistence with published echocardiography studies on Valsalva and Mueller maneuvers. Free breathing and patient's movement did not disturb the scan and image quality with this new technique. This technique might provide new insights into pathophysiologic changes associated for example with preclinical congestive heart failure.

## Funding

None.

**Figure 1:**
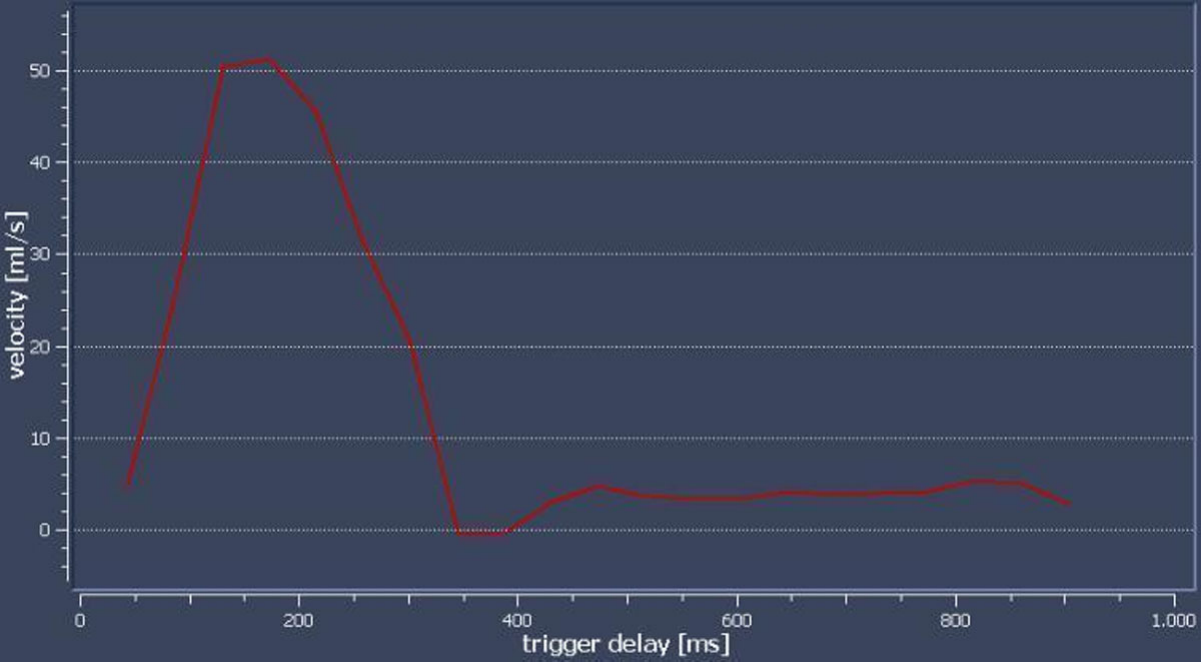
Real-time blood flow in the aorta during Valsalva maneuver.

**Figure 2:**
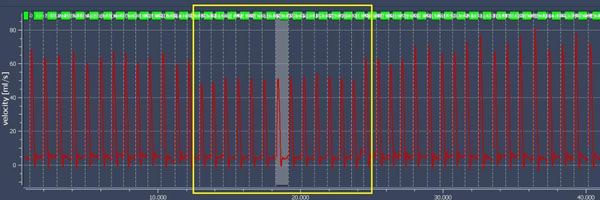
Real-time blood-flow before, during and after Valsalva maneuver. Yellow area: Valsalva maneuver.

